# A novel prognostic prediction tool for postoperative recurrence in patients with stage II/III colon cancer

**DOI:** 10.1186/s40880-019-0397-1

**Published:** 2019-10-01

**Authors:** Jian Ma, Burton B. Yang

**Affiliations:** 1 Department of Urology, Yantai Yuhuangding Hospital, Affiliated with Qingdao University Medical College, Yantai, 264000 Shandong P. R. China; 2 Sunnybrook Research Institute, Toronto, ON M4N 3M5 Canada; 3 Department of Laboratory Medicine and Pathobiology, University of Toronto, Toronto, ON M4N 3M5 Canada

## Main text

Colon cancer, one of the most common cancers in the world [1] and China [2, 3], is typically diagnosed in its advanced stages. However, only a mediocre 20%–30% of these patients can have curative resection, but still, they are at high risk of recurrent disease [4]. Given that recurrent disease is especially associated with poor outcomes, optimizing the therapeutic strategy is paramount. Adjuvant chemotherapy, a standard treatment for stage II/III colon cancer, is often accompanied by a significant increase in survival [5, 6]. Thus, timely adjuvant therapy is primordial for reducing postoperative recurrence risk. To assess the risk of postoperative recurrence, biomarkers of greater prognostic essence than traditional clinicopathology are urgently needed. The emerging role of circular RNAs (circRNAs), a newly discovered type of regulatory RNAs, has shed light on whether these molecules can serve as biomarkers for predicting the postoperative recurrence of colon cancer. circRNAs are characterized by a covalently closed loop that is capable of regulating gene expression at the transcriptional or post-transcriptional levels [7]. They can also bind to proteins and thereby regulate the intracellular signaling pathways [8, 9]. Recent reports have described the roles of circRNAs in the initiation and progression of cancer [10–12], and increasing evidence have demonstrated that circRNAs may serve as molecular biomarkers to facilitate diagnostic and prognostic evaluations [13]. However, the potential of circRNAs for diagnosing and prognosing colon cancer is less clear.

A recent study published in EMBO Molecular Medicine by Ju et al. [14] identified and validated a novel prognostic tool for colon cancer prognosis. They identified differentially expressed circRNAs in patients with or without recurrence through RNA sequencing (RNA-seq) analysis of 20 paired patient tissues, followed by the retrospective analysis of 667 patients with R0-resected stage II/III colon cancer. A four circRNA-based cirScore was generated to classify patients into a high- and low-risk group.

The authors examined 437 differentially expressed circRNAs between the tumour and adjacent normal tissues and 103 differentially expressed circRNAs between recurrent and nonrecurrent tumour tissues. The circRNAs that were differentially expressed between the normal and tumoural tissues had more prominent changes relative to the differences between recurrent and nonrecurrent tumours. The authors then selected 100 significantly upregulated circRNAs to test whether circRNAs could be used as prognostic biomarkers for patients with stage II/III colon cancer. Among the validated candidates, the authors focused on 22 circRNAs, including 10 from the tumour and adjacent tissue groups and 12 from the recurrent and nonrecurrent groups. After quantifying these 22 circRNAs with realtime-PCR, four mutually independent circRNAs (hsa_circ_0122319, hsa_circ_0087391, hsa_circ_0079480, and hsa_circ_0008039) demonstrated strong predictive values for disease-free survival. Analytic results showed that the four circRNA-based risk score (cirScore) outperformed the single circRNAs as prognostic biomarkers. Thus, a four-circRNA-based prognostic model was generated in which the formula $\left( {cirScore = 0.46 \times {\text{Exp}}_{hsa\_circ\_0122319} + \left[ { - 0.386 \times {\text{Exp}}_{hsa\_circ\_0083791} } \right] + 0.293 \times {\text{Exp}}_{hsa\_circ\_0079480} + 0.439 \times {\text{Exp}}_{{{\text{hsa}}\_{\text{circ}}\_0008039}} } \right)$ was developed to evaluate the prognostic predictors. The patients were divided into high- and low-risk groups based on their obtained median value. Patients in the high-risk group had poorer disease-free survival than those in the low-risk group. The cirScore appeared to be a suitable predictor of disease-free and overall survival in the training set after adjusting for baseline clinicopathological factors. The model was also internally and externally validated in testing cohorts using a clinical risk-stratification scheme. With this model, the authors established a nomogram based on the final Cox model for disease-free survival. The nomogram showed good clinical performance for estimating the 3- or 5-year disease-free and overall survival.

The authors also investigated the biological functions of the selected circRNAs in the development of colon cancer. After confirming the circular structure, the authors examined the functions of hsa_circ_0122319, hsa_circ_0079480, and hsa_circ_0087391 since these three circRNAs were highly expressed in the recurrent tissues and in several colon cancer cell lines with high metastatic potential. To explore the biological functions of these circRNAs in vivo, specific recombinant lentivirus-mediated short hairpin RNAs (shRNAs) were constructed and used to decrease endogenous expression of hsa_circ_0122319, hsa_circ_0079480, or hsa_circ_0087391 in colon cancer cell lines. Realtime-PCR, immunoblotting, and RNA-seq analysis were used to verify the silencing effects of these shRNAs. All shRNAs were found to specifically knockdown their corresponding circRNA and had no off-target effects. These circRNAs silencing significantly suppressed cell migration. One of the circRNAs, hsa_circ_0079480, was used in vivo and was found to play important roles in the development of metastatic nodules in the liver and lungs of mice.

Taken together, with a very clear scientific logical flow, the authors identified and validated a four circRNA-based cirScore to improve the prognostic stratification for patients with radically resected stage II/III colon cancer. Supported by well-designed approaches, this proposed cirScore can not only effectively classify patients with stage II/III colon cancer into high- and low-risk groups of recurrence but can also provide additional prognostic value to existing clinicopathological prognosticators for colon cancer patients at this stage. This model is also strongly supported by the validation of the roles of these circRNAs in colon cancer development and progression both in vitro and in vivo. It is anticipated that this approach can be used to develop biomarkers in other types of cancers.

## Data Availability

Not applicable.

## Abbreviations

circRNA: circular RNA
RNA-seq: RNA sequencing
cirScore: circRNA-based risk score
shRNA: short hairpin RNA
